# Mycotic Renal Artery Aneurysm Presenting as Critical Limb Ischemia in Culture-Negative Endocarditis

**DOI:** 10.1155/2018/7080813

**Published:** 2018-05-07

**Authors:** Vy Thuy Ho, Nathan K. Itoga, Tiffany Wu, Ehab Sorial, Manuel Garcia-Toca

**Affiliations:** Department of Surgery, Division of Vascular Surgery, Stanford University, Stanford, CA, USA

## Abstract

Mycotic renal artery aneurysms are rare and can be difficult to diagnose. Classic symptoms such as hematuria, hypertension, or abdominal pain can be vague or nonexistent. We report a case of a 53-year-old woman with a history of intravenous drug abuse presenting with critical limb ischemia, in which CT angiography identified a mycotic renal aneurysm. This aneurysm tripled in size from 0.46 cm to 1.65 cm in a 3-week interval. Echocardiography demonstrated aortic valve vegetations leading to a diagnosis of culture-negative endocarditis. The patient underwent primary resection and repair of the aneurysm, aortic valve replacement, and left below-knee amputation after bilateral common iliac and left superficial femoral artery stenting. At 1-year follow-up, her serum creatinine is stable and repaired artery remains patent.

## 1. Introduction

The visceral arteries are the least common sites for mycotic aneurysm formation, representing ≤1% of intra-abdominal mycotic aneurysms [1, 2]. Risk factors for mycotic renal artery aneurysms (MRAAs) include intravenous drug use, endocarditis [3–5], and renal transplantation [6–8]. These inflammatory aneurysms grow rapidly within days to weeks and are prone to rupture [9]. We describe a patient with critical limb ischemia and culture-negative endocarditis who was found to have a mycotic renal artery aneurysm, which tripled more than its size in three weeks.

## 2. Case Report

**Figure 1:**
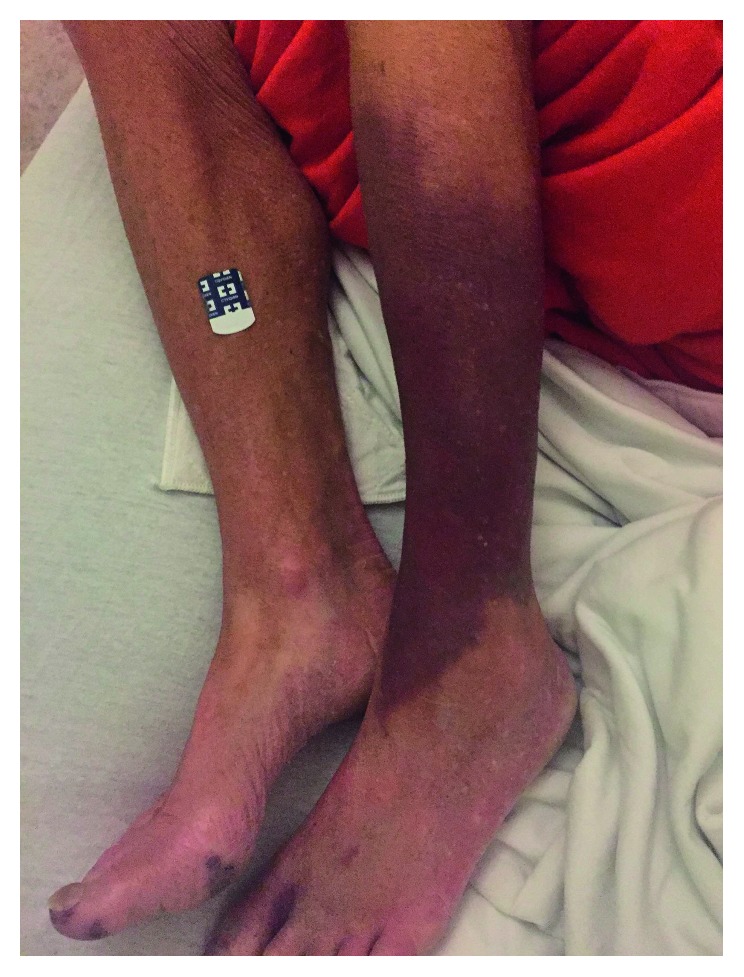
Lower extremity physical exam findings leading to the further workup of embolic and infectious sources, Osler nodes on the right foot, and decreased perfusion of the left leg due to proximal arterial occlusion in the iliac and superficial femoral arteries.

A 53-year-old woman with a history of intravenous methamphetamine abuse, hypertension, tobacco use, and poorly controlled type 2 diabetes presented with 2 weeks of progressively worsening left lower extremity pain and motor deficits. Two weeks prior, she reported hitting her leg against a door while intoxicated. Since then, her left calf had become increasingly painful, swollen, and unable to bear weight. On exam, the left anterior calf and foot were ecchymotic and poikilothermic, and there were multiple tender, raised red lesions consistent with Osler nodes at the plantar surface of the right foot (Figure 1). Ankle brachial indices were unattainable on the left side due to absent waveforms.

**Figure 2:**
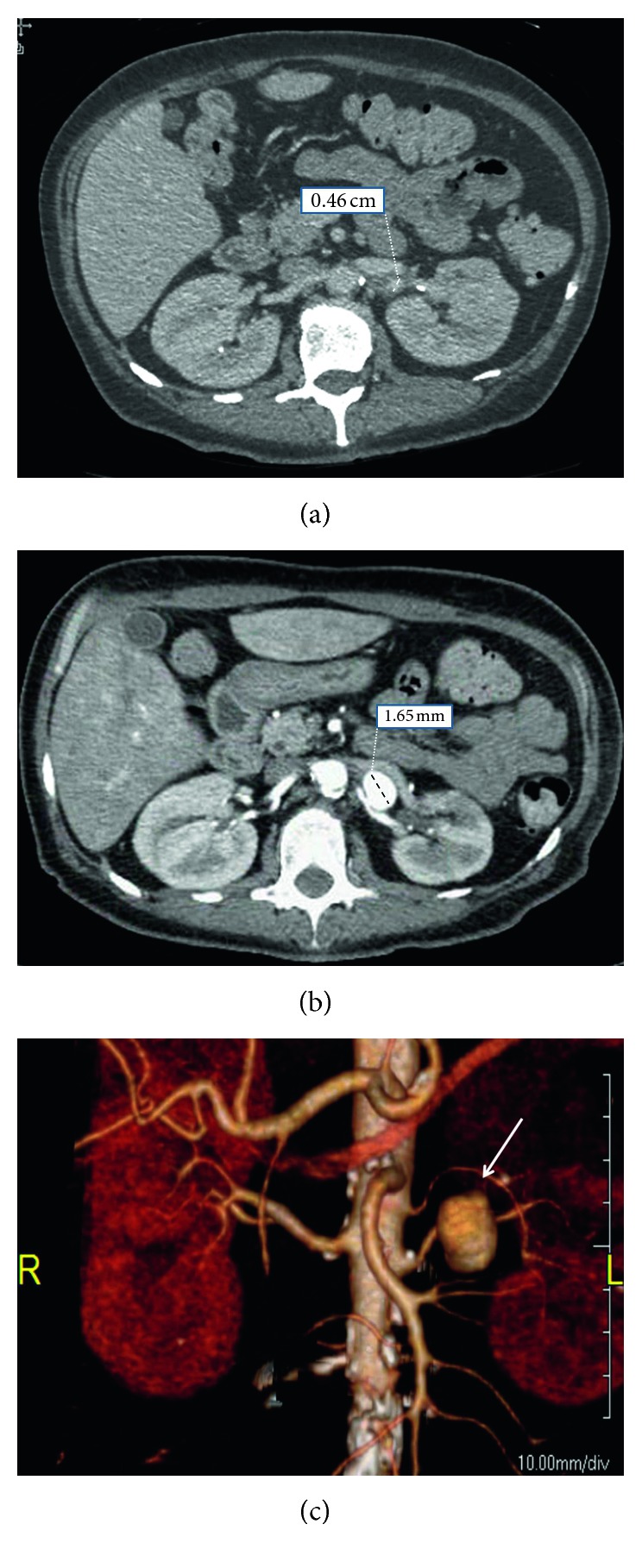
CT axial images demonstrating (a) normal caliber left renal artery 3 weeks prior to presentation, (b) 1.65 cm left renal artery aneurysms, and (c) 3D reconstruction of left renal artery aneurysm (arrow).

The patient also had a one-month history of leukocytosis with negative blood cultures thought initially to be due to a UTI. Her leukocytosis persisted despite oral antibiotic therapy. An abdominal CT scan taken 3 weeks prior to admission showed a normal caliber left renal artery (Figure 2(a)); however, an abdominal CT scan on admission revealed a 1.65 cm left renal artery aneurysm (Figures 2(b) and 2(c)). CTA of the lower extremity demonstrated 50% left iliac stenosis and superficial femoral artery occlusion with popliteal reconstitution and no runoff to the foot. Given concerns for septic emboli, a transthoracic echocardiogram was ordered demonstrating echogenic vegetations on the right coronary cusp of the aortic valve.

**Figure 3:**
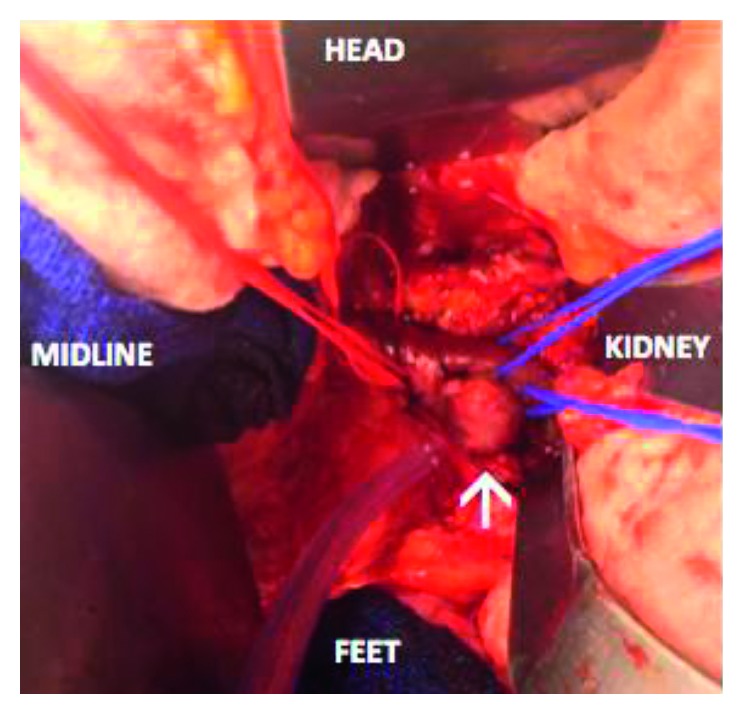
Operative exposure during left renal artery aneurysm (arrow) resection and repair. Proximal and distal control is obtained, and the left renal vein is retracted cephalad.

The renal aneurysm was repaired with a midline transperitoneal approach, and the left gonadal and adrenal veins were ligated for exposure (Figure 3). After proximal and distal control was obtained, the saccular aneurysmal wall was resected and the renal artery was repaired primarily.

Three days later, she underwent angioplasty and stenting of the left common iliac and superficial femoral arteries in an effort to permit healing of a below-knee amputation but ultimately required above-knee amputation. The patient also underwent aortic valve replacement during her admission. Her leukocytosis resolved, and creatinine returned to baseline. At a follow-up visit 24 months postoperatively, the patient's creatinine was at baseline and she had no signs or symptoms of renal insufficiency. Follow-up CT angiogram and renal ultrasound showed a patent left renal artery after repair.

## 3. Discussion

The majority of renal artery aneurysms are asymptomatic [10]. This patient presented with a chronic leukocytosis but did not have abdominal symptoms. Other potential symptoms of MRAAs include fever, hematuria, vague abdominal pain, hypertension, or abdominal bruit [10]. Blood cultures may be negative in up to 27% of patients with mycotic aneurysms [5], further increasing the difficulty of diagnosis. The presence of Osler nodes on the plantar surface of the right foot, leukocytosis, and acute or chronic critical limb ischemia in the form of new foot wounds and motor-sensory deficits raised the concern for endocarditis despite negative blood cultures. With a history of intravenous drug use, arterial embolization, Osler nodes, and echocardiographic evidence of endocarditis, the patient met Duke's criteria for the diagnosis of infective endocarditis [11].

Pathological examination suggests that MRAAs develop from septic emboli that penetrate the vasa vasorum, seeding adventitial inflammation and dilation of the weakened arterial wall [9]. Growth rates of mycotic aneurysms have been reported in the thoracic and abdominal aorta [12–14], but this is the first case report to document the growth rate of a MRAA.

Treatment approaches for MRAAs include open surgical reconstruction, stent- or balloon-assisted coil embolization, and covered stenting. Given the large increase in MRAA size in a short period of time, we chose a surgical intervention, given the lack of reports regarding successful treatment with antibiotics alone. Urgent treatment is recommended as mortality after the visceral mycotic aneurysmal rupture is 80%, with sepsis being the most common cause of death [15, 16]. The benefits of endovascular therapy must be weighed against the risks of graft infection. Case reports have shown success in treating MRAAs with stenting and antibiotics [17–19], although none of these patients had endocarditis at the time of the procedure. Coil embolization is generally reserved for distal or parenchymal lesions [10, 17]. We elected to perform open primary repair to avoid use of prosthetic materials.

## 4. Conclusion

Mycotic renal aneurysms are a rare complication of endocarditis, particularly in the absence of aortic wall involvement or renal transplantation. Our report highlights the importance of clinical workup of embolic sources of critical limb ischemia, as well as documenting the rapid growth of mycotic aneurysms.
